# Paradoxical expression of INK4c in proliferative multiple myeloma tumors: bi-allelic deletion vs increased expression

**DOI:** 10.1186/1747-1028-1-23

**Published:** 2006-10-18

**Authors:** Amel Dib, Timothy R Peterson, Laura Raducha-Grace, Adriana Zingone, Fenghuang Zhan, Ichiro Hanamura, Bart Barlogie, John Shaughnessy, W Michael Kuehl

**Affiliations:** 1 Genetics Branch, Center for Cancer Research, National Cancer Institute, Naval Hospital, Bldg 8, Rm 5101, Bethesda, MD20889-5105, USA; 2 Department of Biology, Masssachusetts Institutes of Technology, Whitehead Institute, 9 Cambridge Center, Rm 359, MA02142, USA; 3 University of Pittsburgh School of Medicine, M218 Scaife Hall, Pittsburgh, PA15261, USA; 4 Myeloma Institute for Research and Therapy, University of Arkansas for Medical Sciences, 4301 Markham St., #816, Little Rock, AR72205-7199, USA; 5 Department of Internal Medicine, Division of Hematology, Aichi Medical University, 21 Karimata, Yazako, Nagakute, Aichi-gun, Aichi 480-1195, Japan

## Abstract

**Background:**

A high proliferative capacity of tumor cells usually is associated with shortened patient survival. Disruption of the RB pathway, which is critically involved in regulating the G1 to S cell cycle transition, is a frequent target of oncogenic events that are thought to contribute to increased proliferation during tumor progression. Previously, we determined that p18INK4c, an essential gene for normal plasma cell differentiation, was bi-allelically deleted in five of sixteen multiple myeloma (MM) cell lines. The present study was undertaken to investigate a possible role of p18INK4c in increased proliferation of myeloma tumors as they progress.

**Results:**

Thirteen of 40 (33%) human myeloma cell lines do not express normal p18INK4c, with bi-allelic deletion of p18 in twelve, and expression of a mutated p18 fragment in one. Bi-allelic deletion of p18, which appears to be a late progression event, has a prevalence of about 2% in 261 multiple myeloma (MM) tumors, but the prevalence is 6 to10% in the 50 tumors with a high expression-based proliferation index. Paradoxically, 24 of 40 (60%) MM cell lines, and 30 of 50 (60%) MM tumors with a high proliferation index express an increased level of p18 RNA compared to normal bone marrow plasma cells, whereas this occurs in only five of the 151 (3%) MM tumors with a low proliferation index. Tumor progression is often accompanied by increased p18 expression and an increased proliferation index. Retroviral-mediated expression of exogenous p18 results in marked growth inhibition in three MM cell lines that express little or no endogenous p18, but has no effect in another MM cell line that already expresses a high level of p18.

**Conclusion:**

Paradoxically, although loss of p18 appears to contribute to increased proliferation of nearly 10% of MM tumors, most MM cell lines and proliferative MM tumors have increased expression of p18. Apart from a small fraction of cell lines and tumors that have inactivated the RB1 protein, it is not yet clear how other MM cell lines and tumors have become insensitive to the anti-proliferative effects of increased p18 expression.

## Background

The RB pathway, which is critically involved in regulating the G1 to S cell cycle transition, has a central role in the regulation of proliferation, senescence, and differentiation in most tissues [1,2]. Cyclin D1, D2, or D3 proteins form complexes with Cyclin D dependent kinases (CDK4 or CDK6), which can then phosphorylate RB-1, and thereby decrease its ability to inhibit E2F transcription factors that increase expression of proteins involved in DNA synthesis. Four INK4 inhibitors (p16INK4a, p15INK4b, p18INK4c, and p19INK4d) can bind to CDK4 or CDK6, thereby inhibiting Cyclin D activation of these kinases.

Cell cycle arrest is tightly coupled to terminal differentiation of B cells into plasma cells, a process that is temporally correlated with an increased expression of p18INK4c [3,4]. Over-expression of transfected p18 restored coupled cell cycle arrest and differentiation in a B-lymphoblastoid cell line that was treated with IL-6. Mice that are null for p18 developed widespread organomegaly, with an increased incidence of pituitary tumors [5]. Lymphoid tissues of these mice exhibited increased cellularity, with B and T lymphocytes showing higher proliferation rates upon mitogenic stimulation, sometimes manifested as lymphoproliferative disorders or lymphoma tumors [6]. Significantly, p18 null B cells did not differentiate into normal plasma cells [7].

One component of the RB pathway is disrupted in most kinds of tumors, primarily by increased expression of CDK4, CDK6, or a CYCLIN D protein, or by inactivation of RB-1 or an INK4 inhibitor (mostly INK4a, or less often INK4b)[1,2]. It appears that the RB pathway often is disrupted by dysregulation of a Cyclin D gene in multiple myeloma (MM), a malignant tumor of plasma cells[8,9]. Several years ago, we reported that five of 16 human myeloma cell lines (HMCL) have bi-allelic deletion of p18INK4c[10]. To help elucidate the prevalence, timing, and significance of p18INK4c abnormalities in multiple myeloma, we have studied 40 HMCL, 261 MM tumors, and 16 normal bone marrow plasma cell samples.

## Results

### Thirteen of 40 human myeloma cell lines do not express p18INK4c

**Figure 1:**
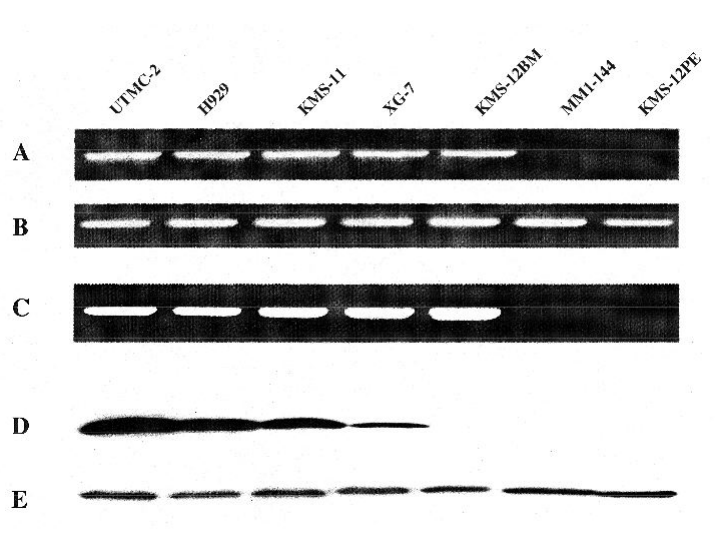
Expression of p18INK4c in representative myeloma cell lines. Results of RNA expression, gene content, and protein expression are shown, with experimental details included in Materials and Results. Agarose gels show: A) a 521 bp exon2-exon3 p18 RT.PCR fragment; B) a 248 bp GAPDH RT.PCR fragment; and C) a 537 bp p18 exon 3 PCR fragment from genomic DNA. Western blots show: D) p18INK4c protein; and E) β-tubulin protein.

Twelve (RPMI-8226; ARP-1; Karpas-620; KMS-12PE; MM-1; OCI-MY1; OCI-MY5; OCI-MY7; OPM-1; OPM-2; SKMM-2; XG-6) of 40 HMCL analyzed have no detectable p18INK4c RNA by an RT.PCR assay (Fig. 1A), and no detectable p18 exon 3 sequences in genomic DNA (Fig. 1C). The remaining 28 HMCL express p18 RNA at levels within an approximately twenty-fold range (Table 1). Western blots (representative results in Fig. 1D) detect an apparently normal p18 protein at a level that parallels the level of p18 RNA in 27 of these HMCL, but no p18 protein was detected in KMS-12BM. Sequence analysis of an RT.PCR product showed that KMS-12BM had a homogeneous p18 sequence, with a TT deletion in codon 21 (CTT) that resulted in conversion of Leu to His, which was followed immediately by a TGA stop codon. Additional sequence analyses of RT.PCR products showed no coding mutations in 13 other HMCL (Table 1).

**Table 1:** p18 and FAF1 RNA in 39 HMCL

HMCL	p18	FAF1	HMCL	p18	FAF1
XG-6#	0.01	0.03	XG-1#	1.05	0.97
OPM-1	0.01	0.03	KMS-18	1.22	1.60
8226	0.02	0.04	KMS-28PE	1.24	0.60
KMS12-PE	0.03	1.08	KHM-11	1.29	1.39
OCI-MY5	0.03	0.01	L363	1.41	1.21
MM-1	0.03	0.07	KMS-11	1.45	0.50
SKMM-2	0.03	0.01	U266	1.48	1.28
ARP-1	0.04	0.02	KMM-1	*1.48	1.38
OPM-2	0.04	0.01	JJN-3	1.58	1.16
OCI-MY1	0.05	0.02	KMS-34	1.68	1.08
OCI-MY7	0.06	0.38	ANBL6#	*1.75	1.61
FR4	*0.30	1.36	EJM	2.06	1.77
JIM-3	*0.34	0.51	FLAM-76#	*2.10	1.66
XG-7#	*0.41	1.29	KMS-28BM	2.30	0.56
KMS12-BM	**0.53	0.74	LP1	*2.43	1.27
ARK	*0.60	1.70	XG-2#	2.46	1.40
MM-S1	*0.72	1.86	PE-1#	2.51	1.36
H1112	*0.80	1.43	H929	*2.91	0.94
SKMM-1	0.97	1.02	UTMC-2	*5.67	1.85
DELTA-47	*1.02	0.89			

Normalized RNA content on HG_U133A Gene Chip (see Methods)

# IL-6 added to culture medium

* normal RT.PCR sequence

** mutant RT.PCR sequence (see Results)

### The consensus minimum deletion region in 13 HMCL selectively targets the p18INK4c gene

**Figure 2:**
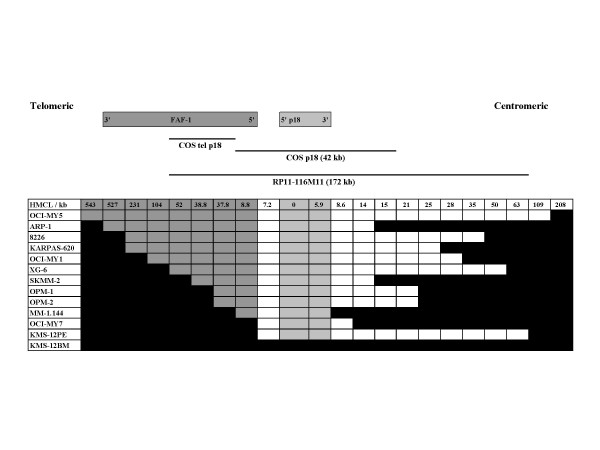
Minimum deletion regions on 1p32.3 for HMCL with bi-allelic deletion of p18INK4c. Pairs of oligonucleotides at the indicated positions (relative to the 5' end of the p18 gene) were used in PCR reactions to identify regions of bi-allelic deletion (black if not bi-allelically deleted). The positions of the FAF1 (dark gray) and p18 (light gray) genes, and FISH probes are shown.

The p18INK4c gene is located on chromosome band 1p32.3, 9 kb centromeric to the Fas associated factor 1 (*FAF1*) gene, which encodes a receptor (TNFRSF6) that is able to mediate apoptosis initiated by interaction with external FAS ligand (TNFSF6) [11]. Since bi-allelic deletion of p18 might affect FAF1, the minimum deletion region was determined for each of the 12 HMCL in which bi-allelic deletion of p18 had been identified (Fig. 2). The minimum deletion region did not involve FAF1 exons in three HMCL (KMS12PE; OCI-MY7: MM.1), but included variable portions of the FAF1 gene in the other nine HMCL. Consistent with these results, FAF1 RNA was not expressed in the nine HMCL that had bi-allelic deletions of some portion of the FAF1 gene. However, with the exception of a very low level of expression in MM-1, FAF1 RNA was expressed in all other HMCL, including KMS-12PE and OCI-MY7 (Table 1). Therefore, p18INK4c is the uniform target of deletion, although it is possible that the associated inactivation of FAF1 could affect the tumor cell phenotype

We found no evidence for deletion "hotspots" since each of the twelve HMCL derived from independent tumors appeared to have unique telomeric and centromeric deletion breakpoint regions (Fig. 2).

The OPM-1 and OPM-2 HMCL, which were derived independently from the same tumor specimen [12], had identical deletion profiles (Fig. 2). Therefore, the deletion most likely occurred in tumor cells in the patient and not during generation or propagation of the cell lines.

### Bi-allelic deletion of p18INK4c is much less prevalent in MM tumors than in HMCL

The expression of p18INK4c RNA was determined for 231 untreated MM tumors, 30 relapsed MM tumors, 33 HMCL, and 16 normal bone marrow plasma cell samples using Affymetrix HG-U95A Gene Chips. The normalized expression of p18 RNA for the 310 samples is summarized in Table 2. Ten of 33 (30%) HMCL have deletion of the p18 gene and normalized p18 RNA values < 0.55, thereby defining a maximum background signal. In contrast, only 28 of 261 (11%) MM samples, and none of 16 normal PC samples have p18 RNA values < 0.55. To directly assess the prevalence of bi-allelic p18 deletion in MM tumors, we did quantitative real time PCR assays to determine the content of p18 genomic DNA in 31 MM tumors, including twelve tumors with p18 RNA values less than the background level of 0.55 (representative results in Table 3). Compared to a reference *CYCLIN D3 *gene, only three tumors (P051, P057-2, P303) had a significant decrease (nine- to fourteen-fold) in p18 gene content compared to placental DNA. None of the other 28 MM tumors, including nine with background values of p18 RNA, had even a twofold decrease in p18 gene content, so that the expression array results alone cannot confirm bi-allelic deletion. Since most purified samples have been estimated to contain greater than 90% tumor cells, it appears that bi-allelic deletion of p18 occurs in no more than 2 to 3% of MM tumors compared to about 30% of HMCL.

**Table 2:** p18 RNA and expression-based proliferation index (PI)

		normalized p18 RNA*			
			
	**# PC**	**≤0.55**	**0.55–1**	**1–2**	**>2**
**PI<1**	16	0	2	14	0
	**# HMCL**				
**PI<1**	0	0	0	0	0
**PI 1–2**	1	0	0	0	1
**PI >2**	32	10	0	3	19
**Total HMCL**	33	10	0	3	20
	**# MM**				
**PI<1**	151	19	89	38	#5
**PI 1–2**	60	3	32	17	8
**PI >2**	50	#6	4	10	#30
**Total MM**	261	28	125	65	43

* Summary of HG_U95A RNA expression data (Methods and Results for details)

# P < 0.00001

### The prevalence of bi-allelic p18 deletion is higher in more proliferative MM tumors

In contrast to highly proliferative HMCL, MM tumors generally have a low proliferation index [13,14]. To assess proliferation in MM tumors, we determined an expression-based proliferation index (PI) from the median expression of twelve genes that are associated with a proliferation signature (see Methods) [9,15]. A context for the PI (which includes Ki67, a standard marker to determine the fraction of cycling cells) is provided by the fact that a PI>2 occurs in 97% of 33 HMCL, 70% of 30 relapsed MM tumors, 13% of 231 untreated tumors, and none of 16 normal plasma cell samples (Table 2). As shown in Table 3, all three MM tumor samples (P051, P057-2, and P303) with bi-allelic deletion of p18 had a PI>2. Only one other tumor sample (P057), derived from the same patient but at an earlier time than sample P057-2, had a PI>2; p18 gene content was nearly two-fold decreased in P057, suggesting the possibility that most tumor cells have mono-allelic deletion of p18, perhaps with some tumor cells have bi-allelic deletion, and also some contamination by normal cells. Significantly, none of eight tumors with a PI <2 and p18 RNA <0.55 had bi-allelic deletion of p18 (Table 3). Including both untreated and relapsed MM tumors, there were 50 tumors with a PI>2. Six of these tumors have background levels of p18, and three of the four tumors analyzed have bi-allelic deletion of p18. Thus, the prevalence of bi-allelic deletion of p18 in more proliferative (PI>2) MM tumors is at least 6% but perhaps 10%, a prevalence that still would be lower than the 30% of HMCL.

**Table 3:** p18INK4c content in MM DNA

**Sample**	**RNA#**	ΔΔCT*	PI**
P303	0.22	3.8	7.25
P057-2	0.31	3.2	3.10
P051	0.42	3.8	2.82
P301	0.41	0.4	0.57
P063	0.41	-0.8	1.33
P408	0.42	-0.5	0.84
P510	0.46	0.7	0.62
P465	0.46	-0.2	0.46
P057	0.50	0.9	3.48
P222	0.51	-1.4	0.94
P015	0.51	0.8	0.69
P237	0.54	0.0	0.49
P504	0.56	-0.7	0.60
P401	0.58	-0.7	0.48
p102	0.59	-0.8	0.70
P266	0.87	0.3	0.46
P277	0.88	0.2	0.82
P208	1.08	0.0	0.91
P067-1	1.17	0.3	0.51
P175	1.34	-0.2	0.80
P250	1.41	0.6	0.73
P163	1.49	-0.5	0.92

# Normalized RNA content by HG_U95A analysis

* real time PCR for p18 DNA content

** expression-based proliferation index

See Materials and Results for details.

### HMCL and more proliferative MM tumors frequently over-express p18

As summarized in Table 2, the normalized expression of p18 RNA was >2 in twenty of 33 (60%) HMCL, and in 43 of 261 (16%) MM tumors, but in none of 16 normal PC samples. The increased expression of p18 in MM tumors was associated with increased proliferation since 5 of 151(3%) MM tumors with a PI<1, 8 of 60 (13%) MM tumors with a PI between 1 and 2, and 30 of 50 (60%) MM tumors with a PI>2 had normalized values of p18 >2. Therefore, unless the p18 gene has been deleted, the expression of p18 RNA usually is increased in HMCL and more proliferative MM tumors.

**Figure 3:**
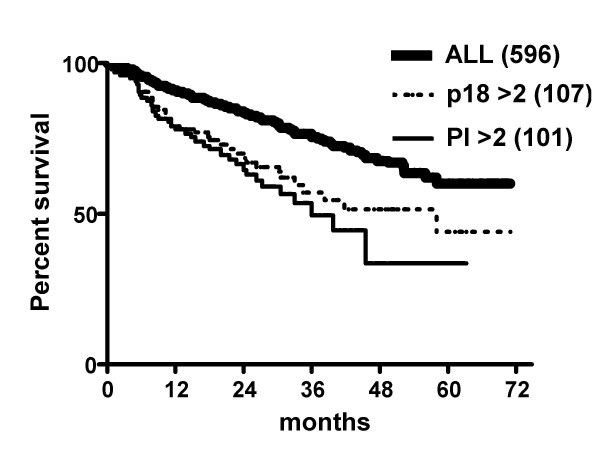
Effect of increased proliferation or increased p18INK4c expression on survival of multiple myeloma patients. Survival data and Affymetrix U133_2.0_Plus RNA expression data was obtained for 596 individuals with MM, including 559 newly diagnosed patients and 37 patients in relapse. Kaplan-Meier curves of overall survival are shown. Compared to the total population, 101 patients with an expression- based proliferation index (PI) >2 had a significantly shorter survival (P < 0.0001), and 107 patients with a normalized p18INK4c expression > 2 also had a significantly shorter survival (P = 0.0005).

A proliferation signature is associated with a poor prognosis in many kinds of tumors, including MM tumors [15,16]. Figure 3 shows a survival curve of 596 individuals with MM tumors, including 559 individuals with untreated tumors and 37 individuals with relapsed tumors[16]. There is a significantly reduced overall time of survival for individuals whose tumors have an expression based proliferation index greater than two (P < 0.0001). Given the strong correlation of a PI>2 with increased expression of p18INK4c, it is not surprising that the overall survival time of individuals with tumors having a normalized expression of p18>2 is also significantly reduced (P = 0.0005). In fact, we confirmed that PI is more important than p18 in determining survival since PI>2 with p18<2 also is associated with a significantly poorer prognosis, whereas p18>2 with PI<2 is not associated with a significantly poorer prognosis.

### Transfected p18 inhibits proliferation of some but not all HMCL

**Figure 4:**
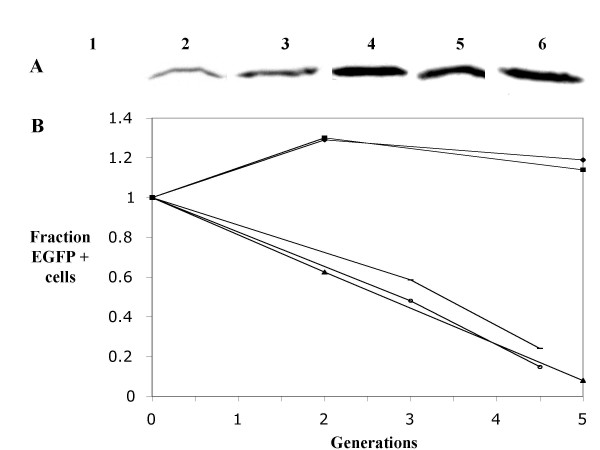
Retrofection of p18 into four HMCL. Representative results are shown for retrofection of four HMCL with pVPG-0 or pVPG-p18 vector. A. Western blot containing extracts from uninfected or infected HMCL. Dilutions of L363 are shown to compare endogenous p18 expression with transfected cells: 1)100 μg uninfected OPM-2; 2)100 μg OPM-2.p18(15% EGFP+); 3)12.5 μg L363; 4)25 μg L363; 5)12.5 μg L363.p18 (puromycin selected); 6) 100 μg of JIM-3. B. Relative growth of infected cells that express EGFP after infection with pVPG-0 (only L363 is shown) or pVPG-p18 (all four HMCL), starting one day after infection (0 generations). The ordinate is the fraction of EGFP cells (normalized to the % EGFP positive cells one day after infection) and the abcissa is the number of generations of the total cell population. Symbols: diamond, L363 (pVPG-0); box, L363; horizontal line, JIM-3; triangle, OPM-2; open circle, KMS-12PE.

To assess the effect of exogenous p18 on proliferation of HMCL that express different amounts of endogenous p18, we used a retroviral vector that co-expresses p18 from the 5' LTR and a puromycin-EGFP fusion product from an internal CMV promoter[17]. We retrofected four HMCL: KMS-12PE and OPM-2 have bi-allelic deletion of p18; JIM-3 expresses a low level of p18; and L363 expresses a high level of p18 (Table 1). Representative results from these experiments are shown in Figure 4. As assessed from Western blots, the level of expression of exogenous p18 in unselected OPM-2 (15% EGFP positive cells) indicated that infected cells express exogenous p18 at a level that is comparable to the endogenous level of p18 in L363 (Fig. 4A). The level of endogenous plus exogenous p18 in puromycin selected (EGFP+) L363 cells appears to be almost twofold higher than the level of endogenous p18 in L363 (Fig. 4A and other results not shown).

To determine the effect of exogenous p18 on growth, the percentage of EGFP positive cells – for both the control and p18 vectors – was determined the day after infection (0 cell doublings), and at two additional times as the entire cell population went through a total of five doublings in the absence of puromycin selection (Fig. 4B). For all HMCL retrofected with the control vector, the fraction of EGFP positive cells increased somewhat during the first two doublings but then remained essentially stable (shown only for L363 in Fig. 4B); this transient period of a relative increase of transfected cells probably is explained by the fact that retroviruses efficiently infect the most healthy, proliferating cells. The growth properties of L363 cells infected with the p18 and control vectors were indistinguishable, indicating no effect of exogenous p18 on cell growth. However, for the other three HMCL that were infected with the p18 vector, the fraction of EGFP positive cells continuously decreased as the population underwent five doublings (Fig 4B). Assuming comparable viability for the transfected and non-transfected cells in each population, we have calculated (Methods) the approximate fractional growth inhibition by exogenous p18 for each of these three HMCL: 79% in a single experiment with OPM-2; 61 and 69% in two experiments with KMS-12PE; 52 and 54% in two experiments with JIM-3.

For L363 and OPM-2, we also did a FACS analysis to determine the DNA content as a measure of cell cycle distribution of EGFP positive cells. For L363 cells examined two population doublings after infection, the fraction of EGFP positive cells with G0/G1 and S DNA content, respectively, was 0.42 and 0.36 with the control vector, and 0.44 and 0.31 with the p18 vector. In marked contrast, for OPM-2 cells examined two population doublings after infection, the fraction of EGFP positive cells with G0/G1 and S DNA content, respectively, was 0.42 and 0.35 with the control vector, but 0.85 and 0.05 with the p18 vector. Therefore, unlike the high p18 expressing L363 where exogenous p18 had no inhibitory effect, the cell cycle and proliferation analyses of p18 null OPM-2 cells are consistent with exogenous p18 markedly inhibiting progression from G1 to the S phase of the cell cycle, thereby causing a substantial inhibition of cell proliferation.

## Discussion

### What are the roles of the INK4a, INK4b, and INK4d genes in MM?

Although polymorphic germline alleles and/or somatic loss of INK4a expression are critical oncogenic factors in murine plasmacytoma tumors [18], the situation is less clear in MM. One individual from a melanoma prone family that carried a germline mutant INK4a gene developed MM, and his tumor cells showed selective loss of the normal INK4a allele [19]. There are no other reports of mutations affecting any of the INK4 genes, but MM tumors with bi-allelic deletion of INK4a, INK4b, and INK4c have been reported [20]. There is scant information about the expression of any of the INK4 genes in MM [21-23]. However, several groups have reported that the INK4a promoter is methylated in 20 to 55% of MM tumors, and more than 80% of extramedullary MM tumors and HMCL [21,24-29]. Some of these same studies also showed a substantial but variable fraction of tumors with methylation of the p15INK4b promoter. The most rigorous study reported that there is methylation of the INK4a promoter in about 20% of purified pre-malignant MGUS (monoclonal gammopathy of undetermined significance) or MM tumor cells, and the INK4b promoter in about 30% of purified MGUS or MM cells [25]. Significantly, a very recent study shows that methylation of p16 occurs at a similar prevalence in MGUS and MM, has no significant effect on expression of p16 RNA, and has no prognostic significance [30]. We also have found little or no expression of p16INK4a RNA in most MM tumors and cell lines, even in the absence of promoter methylation (A. Dib and M. Kuehl, unpublished). Thus it appears that inactivation of p16 expression may not be an oncogenic event in MM, and that p16 methylation may be an epi-phenomenon associated with disease progression. There is virtually no information about the status of INK4d in MM. Although INK4a and INK4b may not be expressed in many (perhaps most) MGUS and MM tumors, presently there is no convincing evidence for oncogenic roles of INK4a, INK4b, or INK4d inactivation in MGUS or MM tumors.

### Why is bi-allelic deletion of p18INK4c more prevalent in HMCL than in MM tumors?

Two possibilities need to be considered in accounting for our finding that nearly one third of HMCL have bi-allelic deletion of p18INK4c, whereas the prevalence is less than 10% for MM tumors that have a comparably high PI. First, since HMCL are preferentially derived from non-hyperdiploid tumors that have a higher prevalence of IgH translocations and other chromosomal structural abnormalities than hyperdiploid tumors[8,14,31], it is possible that these tumors are more likely to have interstitial deletions that involve p18. Second, there might be a stronger selection for bi-allelic deletion of p18 for extra-medullary tumors (and thus HMCL since virtually all of these lines are derived from extra-medullary tumors) compared to intramedullary tumors. In this regard, it is notable that KMS-12BM and KMS-12PE, which were derived at different times from the same patient [32], utilized independent events to fully inactivate p18.

### When does bi-allelic deletion of p18 occur?

It seems likely to be a late progression event since it is more prevalent in MM tumors that have a high proliferation index, which typically occurs late in tumor progression [13,14,33]. More significantly, sequential samples from the same patient showed that only the later sample (P057-2) had bi-allelic deletion of p18 (Results and Table 3).

### Does bi-allelic inactivation of p18 enable increased proliferation?

We do not know if bi-allelic deletion or inactivation of p18 is sufficient for increased proliferation of MM tumors. However, the retroviral mediated expression of p18 in two HMCL (KMS12-PE and OPM-2) that do not express endogenous p18, and in a third HMCL (JIM-3) that expresses very low levels of p18 results in a substantial inhibition of proliferation. Thus, it seems clear that inactivation or decreased expression of p18 represents an important mechanism that contributes to increased proliferation of some MM tumors.

### Disruption of the RB pathway can involve at least two independent events in the same MM tumor

It has been shown that IgH translocations, which are thought to represent very early – if not initiating – events in the pathogenesis of MGUS and MM, directly dysregulate CYCLIN D1 or CYCLIN D3 in about 20% of tumors, and indirectly dysregulate CYCLIN D2 in another 10% or so of tumors [8,17,33]. Dysregulation of one of the CYCLIN D genes may, in fact, represent a virtually universal early event in the pathogenesis of MGUS and MM[8,9]. The low proliferative capacity of MGUS or MM tumors that express a dysregulated CYCLIN D gene is consistent with transgenic models. The high level of expression of transgenic CYCLIN D1 in murine B cells does not seem to perturb normal B cell development and proliferation or lead to tumors unless there is a cooperating oncogenic transgene, such as MYC or activated RAS [34,35]. The bi-allelic inactivation of p18 in HMCL is neither negatively nor positively correlated with the presence of an IgH translocation that directly dysregulates CYCLIN D1, or a MAF gene that indirectly dysregulates CYCLIN D2. Although initially it was thought that only one component of the RB pathway is disrupted in tumors [1], it increasingly is apparent that this is not uniformly true[2]. In addition to examples of tumors in which two different INK4 genes are inactivated, it recently was reported that mantle cell lymphoma, most of which have an IgH translocation that dysregulates CYCLIN D1, become more proliferative when the INK4a/ARF locus is deleted[15]. Thus it appears that for both MM and mantle cell lymphoma, dysregulation of a CYCLIN D gene is an early event associated with low proliferation, whereas inactivation of an INK4 gene can be a late event associated with increased proliferation and a poorer prognosis.

### Why do most HMCL and highly proliferative MM tumors express increased levels of p18?

Compared to normal plasma cells, the level of p18 expression is increased in most HMCL that have not bi-allelically deleted p18 (Table 2 and Results). This increased expression also occurs in 60% of MM tumors that have a PI>2 (comparable to HMCL), whereas MM tumors with a PI <1 rarely have increased expression of p18. Direct support for the conclusion that p18 increases during disease progression has come from expression studies on paired samples taken from patients prior to the initiation of therapy and at relapse. In 22 of 51 (42%) cases, p18 increased an average of 7 fold, and this was associated with an increase in PI (P < 0.0001) (FZ and JS, unpublished). It has been shown that the p18 promoter region has a number of E2F and SP1 binding sites, and that the expression of p18 RNA and protein is up-regulated by E2F1 and SP1 transcription factors [36]. Increased proliferation, which is associated with increased activity of these transcription factors, would be expected to cause increased expression of p18. However, a high level of proliferation with a corresponding high level of p18 suggests that the RB pathway has become insensitive to the inhibitory effect of p18 on CDK-Cyclin D mediated phosphorylation of RB-1. The lack of a growth inhibitory effect of increased p18 expression in retrofected L363 cells (Fig. 4B) supports this inference. The apparent insensitivity of most HMCL to the inhibitory effects of INK4 inhibitors is consistent with the observation that there is a high level of p16INK4a RNA expression in three HMCL (EJM, Flam-76, PE-1) that also express high levels of p18 (Table 1) but not in HMCL that have bi-allelic deletions of p18; whereas 37 of 40 HMCL express little or no p16 RNA (A. Dib, unpublished). The increased expression of p18 in proliferative MM cells is reminiscent of the increased expression of p16INK4a in small cell lung cancer tumors that have inactivated the RB-1 gene[37]. In fact, although we have found that RB-1 protein is expressed in most of the 40 HMCL we have examined, it is not expressed in four HMCL (KMS-18, KMS-28BM, KMS-28PE, U266), providing an explanation for insensitivity to the expression of high levels of p18 in these cell lines (A. Zingone, unpublished)[38]. Others have shown that RB-1 protein is not expressed in about 5% of a limited number of MM tumors that have been analyzed[39,40]. Thus, inactivation of RB-1 apparently accounts for only a small fraction of HMCL or proliferative MM tumors that express increased levels of p18. Recently, it was suggested that increased proliferation of MM tumors is due to increased expression of CDK4 and/or CKD6 [41]. However, there was only a minimum of data supporting this hypothesis, and we could not demonstrate a significant correlation of increased CDK4 or CDK6 RNA expression with an increased expression based proliferation index in primary MM tumors. Finally, in comparing the gene expression profiles of HMCL that express high levels of p18 vs HMCL that have bi-allelic deletion of p18, we do not observe significant differences in gene expression for these two groups (data not shown).

## Conclusion

It seems apparent that increased proliferation of MM tumors sometimes is related to inactivation or decreased expression of p18INK4c. However, apart from a small fraction of tumors that inactivate RB-1, it remains to be determined how most MM tumors are able to overcome the anti-proliferative effect of a high level of p18INK4c.

## Methods

### Cell culture

The 40 HMCL in this study included Karpas-620 and the 39 HMCL in Table 1. [42].

### PCR analyses

Procedures for isolation of total RNA and DNA, synthesis of cDNA, PCR reactions, gel electrophoresis, and isolation of fragments for sequencing were described previously [42]. The expression of p18 RNA (reference sequence NM001262) was determined using a cDNA template with nt 1204–1224 (exon 2) and nt 1722-1698 (exon 3) as primers. To determine the sequence of the p18 coding region (nt 1216–1722), nt 1164–1185 and nt 1784-1765 were used as primers. Exon 3 of p18 was amplified from genomic DNA using nt 1477–1498 and nt 2011-1991 as primers.

### p18 Western blotting

Total protein lysates (50 μg) were resolved by electrophoresis on 12% polyacrylamide/SDS gels, and transferred to nitrocellulose membranes. Antibody SC-865 (Santa Cruz Biotechnology) was used to detect p18, and AA2 antibody (Upstate) was used to detect β-tubulin [42].

### p18INK4c deletion map

A sequence contig that included the p18INK4c gene was obtained from NCBI. After exclusion of repetitive sequences, PCR primer pairs encompassing the p18 gene and 750 kb of flanking sequences were designed and validated using a placental DNA template.

### Real-Time quantitative PCR on genomic DNA

These analyses were performed using the ABI Prism 7700 Sequence Detection System (Applied Biosystems, Foster City, CA). Primers-probe sets (Synthegen, Houston, TX) included: AGGGCCACCGAACTGCTACT, GTGGCTGGATGCTGACGTG, and (FAM)CGCCTTTGGCGATCATCTTTTAACCCT(TAMRA) for p18INK4c; and TCACAAAGCCCAAGGGATCT, AAAGGCGCTGCTGGTCAG, and (JOE)CCGGCCAGCCATGTCTGCATT(TAMRA) for CYCLIN D3. Multiplex PCR reactions were carried out with the TaqMan Universal PCR Master Mix, AmpErase UNG (Applied Biosystems, Branchburg, NJ, USA). The PCR conditions were as described by the manufacturer. Triplicate or quadruplicate experiments were done for each sample, and the threshold cycle values were averaged using the Sequence Detector software 1.7 (Applied Biosystems). For each sample, the average Ct value for the endogenous CYCLIN D3 reference was subtracted from the average Ct value for p18, to yield the ΔCt. The placental DNA ΔCt was subtracted from the ΔCt for each sample to give the corresponding ΔΔCt for each sample.

### Affymetrix gene expression analyses

Isolation of purified normal or tumor plasma cells, and profiling of RNA was performed as described previously [43,44]. Gene-expression intensity values, which were obtained with the use of Affymetrix version 5.01 MAS software, were log transformed and normalized to the median of all genes for each sample, and analyzed using GeneSpring 5.1 (Silicon Genetics, Redwood City, CA). The expression of a specific gene was normalized to the median expression of that gene in the 40 HMCL analyzed on HG-U133 Set Affymetrix Gene Chips, in the 310 samples analyzed on HG-U95A Affymetrix Gene Chips, or in the 596 tumors analyzed on HG-133-2.0-Plus chips. The average of the normalized values for two p18INK4c (204159_at; 211792_s_at) or three FAF1 (218080_x_at; 222511_x_at; 224217_s_at) U133 probe sets is shown in Table 1. The RNA from 10 HMCL and 10 primary MM tumors that cover the full range of p18 expression were analyzed by RT.PCR assays (Applied Biosystems, Foster City, CA) to validate the HG-U95A and HG-U133 quantitation of p18 RNA content. For the U95A data set (Tables 2 &3), an expression-based proliferation index (PI) was calculated using the median value of twelve genes associated with a proliferation signature (9, 15). For 596 tumors analyzed on Affymetrix HG_U133_2.0_Plus Gene Chips, the 12 probe sets used to calculate a PI included: 214710_s_at; 212022_s_at; 202503_s_at; 206102_at; 201897_s_at; 201292_at; 202954_at; 204026_s_at; 204033_at; 204444_at; 203418_at; 202870_s_at. The proliferation index, calculated from the median expression of these twelve genes, was strongly correlated with expression of each gene (R > 0.85 for each gene).

### p18 retrofection[17]

Normal p18 cDNA encompassing 4 bases upstream of the ATG and 12 bases downstream of the termination codon (nt 1212–1736) was PCR amplified from EJM cDNA, cloned into BamHI and XhoI sites to generate pVPG-p18, and the coding sequence verified by sequencing. For both the p18 and control vector, infection efficiencies, estimated from the fraction of EGFP positive cells one day after infection, were approximately 15 to 20% for each of the HMCL except JIM-3, which was 6 to10%. Assuming equal viability of EGFP positive (transfected) and EGFP negative cells without puromycin selection, a decreased fraction of EGFP positive cells with successive population doublings provides a measure of fractional growth inhibition (i), using the equation: n log [(2 - i)/2] = log (%EGFP cells after n population doublings/%EGFP cells initially).

### Flow cytometry

The fraction of EGFP positive cells was determined with a FACSort (BD Biosciences). For cell cycle analysis, cells were stained with Hoechst 33342 at a concentration of 5 μg/ml for 90 minutes at 37°C. An LSR II BD (BD Biosciences) and FACSDiVa software was used to determine the DNA content of individual cells, including EGFP positive and negative cell populations[45]. The fractions of cells in G0/G1, S, and G2/M were calculated using ModFit software (Becton Dickinson).

### Survival data

Survival data and RNA expression data for purified MM tumor cells analyzed on HG_U133_2.0_Plus Gene chips was obtained on 596 patient with MM, including 549 patients with newly diagnosed MM and 37 patients with relapsed MM, as described in detail elsewhere[16].

## Abbreviations

EGFP, enhanced green fluorescent protein; HMCL, human myeloma cell line; MM, multiple myeloma; PI, expression-based proliferation index; MGUS, monoclonal gammopathy of undetermined significance

## Competing interests

The author(s) declare that they have no competing interests.

## Authors' contributions

AD performed molecular biology analyses

TRP performed molecular biology analyses

LRG performed sequencing and transfection

AZ performed molecular biology studies and flow cytometry

FZ performed and analyzed expression arrays

IH performed and analyzed expression arrays

BB provided patient samples

JS participated in design of study and supervised expression array analyses

WMK conceived of the study, participated in its design and coordination, and helped draft the manuscript
